# Adipose-Derived Stromal Cells and Mineralized Extracellular Matrix Delivery by a Human Decellularized Amniotic Membrane in Periodontal Tissue Engineering

**DOI:** 10.3390/membranes11080606

**Published:** 2021-08-10

**Authors:** Dilcele Silva Moreira Dziedzic, Bassam Felipe Mogharbel, Ana Carolina Irioda, Priscila Elias Ferreira Stricker, Maiara Carolina Perussolo, Célia Regina Cavichiolo Franco, Hsueh-Wen Chang, Eltyeb Abdelwahid, Katherine Athayde Teixeira de Carvalho

**Affiliations:** 1 Advanced Therapy and Cellular Biotechnology in Regenerative Medicine Department, The Pelé Pequeno Príncipe Research Institute, Child and Adolescent Health Research & Pequeno Príncipe Faculties, Curitiba, Paraná 80250-060, Brazil; dilceledz@gmail.com (D.S.M.D.); bassamfm@gmail.com (B.F.M.); anairioda@gmail.com (A.C.I.); priscilaeferreira@gmail.com (P.E.F.S.); perussolo10@gmail.com (M.C.P.); 2 Cell Biology Department, Federal University of Paraná, Curitiba, Paraná 81530-000, Brazil; crcfranc@terra.com.br; 3 Department of Biological Sciences, National Sun Yat-sen University, Kaohsiung 80424, Taiwan; hwchang@faculty.nsysu.edu.tw; 4 Feinberg Cardiovascular Research Institute, Feinberg School of Medicine, Northwestern University, Chicago, IL 60611, USA; eltyeb.abdelwahid@northwestern.edu

**Keywords:** bone tissue engineering, stem cell transplantation, adipose-derived stromal cells, human amniotic membrane, periodontal regeneration

## Abstract

Periodontitis is a prevalent disease characterized by the loss of periodontal supporting tissues, bone, periodontal ligament, and cementum. The application of a bone tissue engineering strategy with Decellularized Human Amniotic Membrane (DAM) with adipose-derived stromal cells (ASCs) has shown to be convenient and valuable. This study aims to investigate the treatments of a rat periodontal furcation defect model with DAM, ASCs, and a mineralized extracellular matrix (ECM). Rat ASCs were expanded, cultivated on DAM, and with a bone differentiation medium for four weeks, deposited ECM on DAM. Periodontal healing for four weeks was evaluated by micro-computed tomography and histological analysis after treatments with DAM, ASCs, and ECM and compared to untreated defects on five consecutive horizontal levels, from gingival to apical. The results demonstrate that DAM preserves its structure during cultivation and healing periods, supporting cell attachment, permeation, bone deposition on DAM, and periodontal regeneration. DAM and DAM+ASCs enhance bone healing compared to the control on the gingival level. In conclusion, DAM with ASC or without cells and the ECM ensures bone tissue healing. The membrane supported neovascularization and promoted osteoconduction.

## 1. Introduction

Periodontitis is one of the most prevalent infectious diseases, predominantly caused by anaerobic bacteria, characterized by inflammation and the destruction of protective tissues and dental support, alveolar bone, cementum, and periodontal ligament [1]. The immuno-inflammatory response to this pathological condition, which develops in response to the chronic presence of a biofilm, bacterial plaque, and calculus, destroys the structural components of the periodontium, leading to the clinical signs of periodontitis and even tooth loss [1,2]. Healing observed after conventional treatments is considered periodontal repair, with the formation of a long junctional epithelium accompanying the root surface because the apical migration of gingival epithelial cells into the defect area obstructs regeneration [3]. Periodontal tissue regeneration relies on the undisturbed formation of a new connective tissue attachment to the cementum covering the root and bone, embedded as Sharpey’s fibers, with periodontal ligament collagen fiber bundles spanning perpendicularly between the alveolar bone and cementum, recuperating the original architecture and function of these tissues [3,4].

Diverse forms of treatment, seeking to overcome the limitation of conventional treatments, have employed strategies to stimulate the physiological response or offer elements that naturally participate in tissue formation, healing, and organogenesis. Tissue engineering strategies, using cellular sources [5,6,7,8], inducing factors such as the Bone Morphogenetic Protein [9] and Transforming Growth Factor [10], have been investigated in periodontal regeneration.

Mesenchymal stem cells (MSCs) may provide beneficial effects on periodontal healing being isolated from many adult sources, including adipose tissue [11], which is more readily available. Preclinical studies have revealed the improvement of bone and periodontal healing with the association of adipose-derived stromal cells (ASCs) with different carrier materials: Platelet Rich Plasma [12,13], Collagen [14,15], Ceramics [16,17], and Synthetic polymer [18,19]. There is a reasonable level of evidence that adipose-derived cells may contribute towards periodontal regeneration [20].

The amniotic membrane, the innermost layer of the placenta, can be collected after informed ethical consent and without ethical debate since this material is usually discarded. This tissue is immune-privileged, contains immunoregulatory factors [21], with tissue engineering applications [22], and has been recognized as an alternative to other commercially available membranes for bone regeneration [23]. The applications of the amniotic membrane include fresh, cryopreserved, lyophilized, and commercialized products, besides the resulting biomaterial from de-epithelialization and decellularization [22]. In vitro studies have suggested the application of decellularized amniotic membrane (DAM) as a substrate for cell adhesion, proliferation, and differentiation, providing a matrix for cell invasion [24], facilitating osteogenic cell differentiation [25], as a scaffold for MSCs [26], and as a collagenous carrier for cell transplantation [27]. DAM has been investigated in vivo for guided bone regeneration [28], assessing different amniotic membrane preservation methods [29]. The association of DAM with different cell sources has been examined in bone defects with periodontal ligament cells [30], osteoblasts [31], and ASCs [32,33].

Preclinical studies with the application of DAM in dentistry have been developed in bone with ASCs [34], in oral mucosa with oral epithelial cells [35], in periodontal tissue engineering with periodontal ligament cells [36,37], and with a dental pulp-derived cell sheet [38]. Amnion and its products have been used in clinical trials of periodontal diseases treatment and surgery [39] and clinically to treat gingival recessions [40]. Autologous transplantation of epithelial cells associated with DAM has been used to treat mucosal defects [41].

This study investigated a new strategy to graft the bone and periodontal lesion area with amniotic membrane, adipose-derived stromal cells, and a mineralized extracellular matrix deposited in vitro to stimulate bone deposition in the defect, simulating the application of an allogeneic or even an autogenous graft prepared ex vivo.

## 2. Materials and Methods

### 2.1. Experimental Design and Ethics

The experimental design consisted of in vitro preparation of a decellularized human amniotic membrane for transplantation of adipose-derived stromal cells and mineralized extracellular matrix, followed by the treatment of a rat periodontal furcation defect model (the region where the dental roots divide) with the prepared grafts and observations by micro-computed tomography and histology. Human Amniotic Membrane decellularization, adipose-derived stromal cells differentiation potential, and characteristics of the deposited mineralized extracellular matrix onto and into decellularized Human Amniotic Membrane have been published [32]. The Research Ethics Human Committee of Pequeno Príncipe Faculties and co-participating institutions approved human amniotic membrane collection numbered 659.204 and dated 9 March 2015 (Curitiba, Brazil); informed consent was received from two pregnant donors undergoing natural delivery. Animal experimentation complied with the ARRIVE guidelines, the national guidelines for the care and use of laboratory animals, and followed the protocols approved by the Animal Care Ethics Committee of Pequeno Príncipe Faculties, numbered 018-2012 and dated 21 October 2013 (Curitiba, Brazil).

### 2.2. Amniotic Membrane

Immediately after placental expulsion, the amniotic membrane was separated manually from the chorion and washed with Phosphate Buffered Saline (PBS 2% Penicillin/Streptomycin). The amnion was handled within a laminar flow Class II Biosafe, cut into 15 × 15 cm^2^ pieces, separated into T75 flasks with 200 mL decellularization solution 0.1% Sodium Dodecyl Sulphate (SDS), and placed on a horizontal shaker at 120 rpm for 24 h. Gentle cell scraping and five washing cycles with PBS followed the decellularization with SDS. Membranes were cut into 8 mm diameter disks with a surgical punch, placed on culture dishes, kept in PBS, and exposed to ultraviolet light for 1 h inside a laminar flow hood. The decellularized amniotic membrane (DAM) disks were incubated in complete regular cell culture supplemented medium (Dulbecco’s modified Eagle’s medium-F12, supplemented with 10% fetal bovine serum and 1% streptomycin/penicillin) in standard cell culture conditions of 5% CO_2_ in air at 37 °C for 72 h, before cell cultivation or animal transplant. Amniotic membrane was prepared for histological observations by fixation with 10% natural buffered formalin, dehydration, paraffin embedding, cross-sectioning, and staining with Hematoxylin and Eosin (H&E).

### 2.3. Adipose-Derived Stromal Cells

The methodology used to collect and isolate adipose tissue cells from four 8-week-old male Wistar rats (mean weight 300 g) was adapted from previous studies [42,43]. Inguinal adipose tissue was collected after intraperitoneal anesthesia with 50 mg·kg^−1^ ketamine and 6.6 mg·kg^−1^ xylazine, followed by euthanasia through intracardiac administration of Thiopental 75 mg·kg^−1^. Adipose tissue was handled within a laminar flow Class II Biosafe, washed with PBS, macerated with two scalpels, and digested with 0.075% collagenase type I in PBS during incubation at 37 °C, under agitation for 30 min. Enzymatic activity was inactivated by an equal volume of cell culture medium, Dulbecco’s Modified Eagle’s Medium (DMEM), with 10% Fetal Bovine Serum (FBS). Tissue centrifugation at 1200 rpm for 10 min resulted in a cell pellet, which was resuspended in PBS, filtered through a 100 μm sieve, centrifuged again, resuspended in cell culture medium, and the stromal vascular fraction (SVF) was cultivated at the initial passage at 1 × 10^5^ cells/cm^2^ in T25 culture flasks. The cell culture medium was changed every 72 h. After reaching 80% of cell semiconfluency, adipose-derived stromal cells (ASCs) were transferred with 0.25% trypsin/0.1% EDTA incubation, expanded from a plating density of 1 × 10^3^ cells/cm^2^, and cryopreserved in 80% FBS, 10% medium, and 10% dimethyl sulfoxide.

### 2.4. Transplantation Materials

Four groups of materials were used for animal transplantation in the treatment of furcation lesions in rats: T1 (DAM, only membrane), T2 (DAM+ASCs), T3 (DAM+ECM), and T4 (DAM+ECM+ASCs). ASCs 2.5 × 10^4^ cells/cm^2^ were cultivated over the 8 mm diameter DAM disks and stretched onto 12-well plates. Four days before surgery, cells were associated with DAM in treatment groups T2 and T4. Preparation of DAM with ECM (T3 and T4) required cell culture and osteogenic differentiation in advance to simultaneously heal the different groups. Disks of DAM were associated initially with 2.5 × 10^4^ cells/cm^2^ ASCs and a regular cell culture medium for cell proliferation. After cell semiconfluency observation, osteogenic differentiation of ASCs was induced with Rat Osteoblast Differentiation Medium (Cell Applications Inc., San Diego, CA, USA) for four weeks.

Cell and extracellular matrix distribution was observed after 2.5% glutaraldehyde fixation with histochemical staining with Giemsa and Alizarin red, respectively. Primary antibodies to Osteopontin (OPN, Rabbit polyclonal anti osteopontin ab8448, ABCAM, Cambridge, UK), followed by Alexa Fluor 488-conjugated secondary antibodies (Goat anti-rabbit IgG ab 150077, ABCAM, Cambridge, UK) were used for protein immunolabeling and Hoechst H3569 (Invitrogen, Eugene, OR, USA) for nuclear staining.

### 2.5. Surgical Procedure

Surgery to prepare for class II furcation periodontal defects was conducted in twenty-five 8-week-old male Wistar rats, with the same anesthetic protocol described before. A similar defect model was used by other authors [36]. An incision following the palatal gingiva insertion of the upper first molar exposed the furcation region. Defect preparation between the roots initiated with a carbide drill n.1/4 adapted on handpiece at low speed (500 rpm) with continuous saline solution irrigation to remove bone from the inter radicular region, creating bilateral defects with 2 mm depth from the amelocemental junction and 1.5 mm mesiodistally. Exposed dental roots were smoothed with a periodontal curette and the defects were washed with saline solution. The prepared defects received one treatment from the membrane groups studied (T1, T2, T3, or T4), with T0 (empty defect) on the contralateral side of each animal. The membrane was not applied over the bone defect as a space-maintaining barrier in guided tissue regeneration but implanted as a filling material inside the defect as a bone grafting material. Soft tissues were re-approximated and sutured with 7.0 polypropylene. Animals received analgesics for three days and were fed in powered food for one week to minimize the risk of opening the wounds.

### 2.6. Micro-Computed Tomography

After four weeks of healing and animal euthanasia with the same protocol described before, tissue blocks of both maxilla were removed and kept in 4% formaldehyde in PBS buffered to pH 7.2 for at least one week before examination by micro-computed tomography (micro-CT) at Laboratório de Análises de Minerais e Rochas (LAMIR, Curitiba, Brazil) in a Sky Scan 1172 (Bruker micro-CT, Kontich, Belgium) with 12.89 μm pixel size, 1336 × 2000 pixel resolution, 1.1 s exposure, 89 Kv, and 112 μA. The area of bone deposited between the roots (tangent lines to the roots), measured using the Image J analysis program, was used to quantify the percentage of defect bone healing in five selected tomographic sections, 1 mm from the root bifurcation: Level A (gingival, most superficial), Level B (155 μm from A), Level C (medium, 310 µm from A), Level D (465 µm from A), and Level E (apical, 620 μm from A).

### 2.7. Histology

Tissue blocks were decalcified in 10% EDTA, embedded in paraffin, sections were stained with H&E, Picrosirius Red (PSR), and prepared for immunohistochemistry with Anti-osteocalcin (goat polyclonal IgG, 100 μg/mL), Santa Cruz Biotechnology, Dallas, TX, USA), detected by binding Horseradish peroxidase-conjugated secondary antibodies.

### 2.8. Statistical Analysis

Statistical analysis of differences between treatments on different horizontal levels was performed using the Repeated Measures of ANOVA followed by Student–Newman–Keuls test. A 0.05 level of probability was considered the significance level to reject the null hypothesis that there is no significant difference between treatments.

## 3. Results

### 3.1. Transplantation Materials

**Figure 1:**
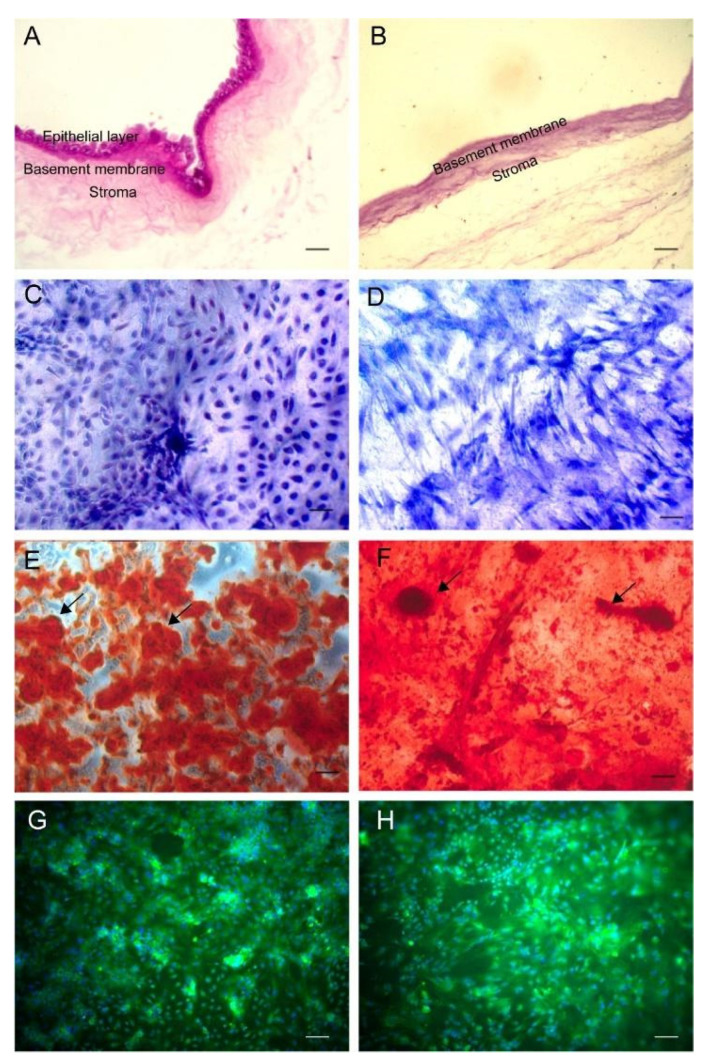
Amniotic membrane histological cross-sections of the intact amniotic membrane with epithelial cell layer (arrow, (**A**)) and after decellularization with 0.1% Sodium Dodecyl Sulphate as a collagenous scaffold (**B**). Representative areas of adipose-derived stromal cell (ASCs) cultures on polystyrene (**C**,**E**,**G**) and decellularized amniotic membrane (DAM) (**D**,**F**,**H**). ASCs confluence in the presence of supplemented medium (**C**,**D**). The osteoinduction medium stimulated the deposition of the mineralized extracellular matrix observed as red calcified deposits (arrows). Osteopontin expression was evidenced by green fluorescence, as agglomerates on polystyrene (**G**), and a diffuse distribution on DAM (H). H&E stain (**A**,**B**); Giemsa stain (**C**,**D**); Alizarin red stain (pH 5.5) (**E**,**F**); green-fluorescent Alexa Fluor 488, Hoechst blue-fluorescent nuclear staining (**G**,**H**); objectives ×20 (**A**–**H**); scale bars 100 µm (**A**–**H**).

The human amniotic membrane decellularization process removed the amniotic-derived epithelial cells and mesenchymal stromal cells effectively, resulting in a collagenous acellular scaffold (Figure 1A,B). ASCs collected from rats and cultured onto polystyrene and DAM adhered and proliferated on both substrates in a complete regular cell culture supplemented medium, preserving a fibroblastic shape (Figure 1C,D). Cell culture with an osteoinduction medium for four weeks stimulated the cell differentiation and deposition of the mineralized bone-like extracellular matrix (Figure 1E,F) as calcified globular accretions rich in Osteopontin (Figure 1G,H). A mineralized matrix was observed on the DAM surface and inside the DAM collagenous scaffold. The deposition of the mineralized ECM on DAM was not uniform on the entire DAM disks and caused a slight increase in volume and stiffness in certain areas.

### 3.2. Bone Healing

**Figure 2:**
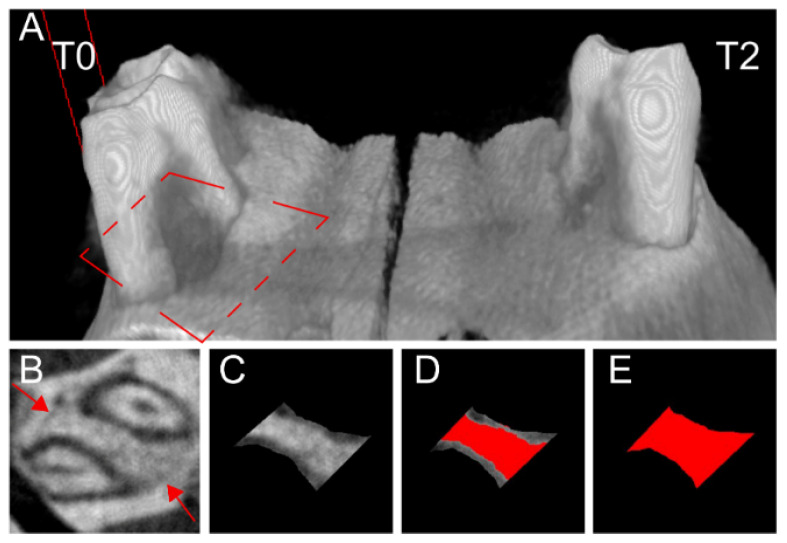
Micro-computed tomography (Micro-CT) images after four weeks of periodontal furcation defect healing. Reconstructed three-dimensional image of the defects with a darker area between the left molar roots without treatment (T0), compared to the defect on the right side treated with decellularized amniotic membrane and undifferentiated adipose-derived stromal cells (T2) (**A**). A dashed rectangle indicates horizontal level orientation for bone healing quantification (**A**). Micro-CT image of a left-side furcation defect treated with T2 (**B**–**E**). The lower bone density of the newly deposited tissue can be observed, also demarcated by the defect preparation perimeter (arrows, (**B**)). Image processing for quantification using Image J software on level D, between medium and apical level, with 77% of bone tissue (**C**–**E**). The area between the roots was restricted with tangent lines, and the roots were contoured and excluded from the image (**C**). The area of mineralized tissue (red in (**D**)) was subtracted from the total area (red in (**E**)) to calculate the percentage of bone healing.

One week after the surgery, the gingival tissue was closed and the sutures fell out. After four weeks of the healing, the tissue blocks were collected and scanned by micro-CT (Figure 2). Measurements of bone density in healing area of rat periodontal furcation defects were performed with the same parameters on five horizontal levels, 1 mm from root bifurcation (Figure 2).

Five animals with clinically deficient mucogingival closure were excluded from the statistical analysis. The possible increase in stiffness and volume of the membranes with ECM (T3 and 4) may explain the deficient mucogingival closure, which caused the exposure of the membranes to the oral cavity. ANOVA with repeated measures was used to compare all group means; those subjected to the different treatments and control (T0–4) and the healing response to each of these treatments and control were compared on each of the five horizontal levels, from gingival to apical (A–E levels from root bifurcation) with Newman–Keuls multiple comparison test (Table 1). Bone healing density was more pronounced on the treatments with a membrane (T1) and the membrane with undifferentiated ASCs (T2) compared to control on Level A (Table 1).

**Figure 3:**
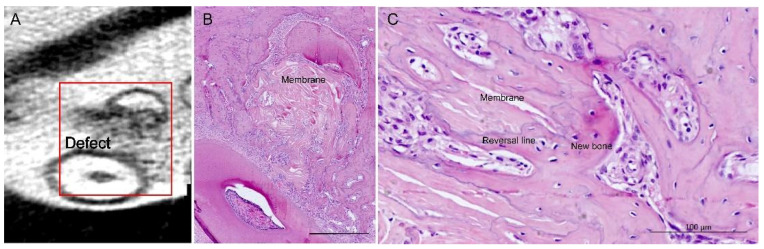
Healing of a defect in a right maxillary molar furcation defect, transplantation of decellularized amniotic membrane associated with undifferentiated adipose-derived stromal cells (T2). Micro-computed tomography image of bone healing between the two molar roots (**A**). Histological section of the same rectangular area in (**A**), with membrane filling the defect between dental roots, orientating bone deposition; in B, osteoconduction by the decellularized membrane and in C demonstrated by vascularization between the membrane folds and distinctive, purple-stained reversal line delineating new bone deposition on loose collagenous fibers of the grafted membrane. H&E stain (**B**,**C**); scale bars 1 mm (**A**), 500 µm (**B**), 100 µm (**C**).

The decellularized amniotic membrane inside the alveolar submucosa gingival connective tissue, associated or not with cells and mineralized extracellular matrix, presented no inflammatory infiltrate or encapsulation. Histological observations demonstrated that the deposition of bone originated from the margins, the remaining alveolar bony housing (Figure 3). The amniotic membrane did not interfere with the deposition of new bone. The membrane directed the deposition of the bone matrix on its collagenous matrix, providing a foundation for bone growth into the defect. Vascularization was observed between the folds of the membrane. Cement lines, boundaries between different periods of bone deposition, observed on the surface of the bone drilled during the surgical procedure, provided the distinction between old and newly deposited bone, which also coincided with the membrane osteoconduction: bone deposition onto and into the amniotic fibrous matrix (Figure 3).

### 3.3. Periodontal Healing

**Figure 4:**
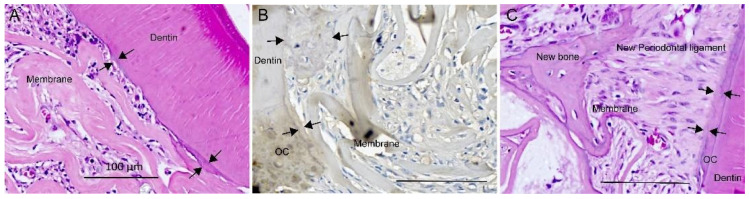
Newly deposited cementum-like matrix (between arrows) observed as a thin metachromatic adherent layer on exposed dentin (**A**,**B**) and exposed old cementum (OC) (**B**,**C**), as thin acellular cementum layer and thick cellular cementum (**B**). Organization of new periodontal ligament (**C**) with periodontal ligament tissue fibers oriented perpendicular to cementum between cementum-like matrix and the newly deposited bone matrix in the niche created by collagenous fibers of the amniotic membrane (**C**). Transplantation of decellularized amniotic membrane (DAM) (T1) (**A**), DAM associated with extracellular matrix and undifferentiated adipose-derived stromal cells (T4) (**B**), DAM associated with extracellular matrix (T3) (**C**). H&E stain (**A**,**C**); immunohistochemistry anti-osteocalcin (**B**); scale bars 100µm (**A**–**C**).

The amniotic membrane from all four treatments tested did not affect periodontal healing by suppressing the new cementum and periodontal ligament. A newly deposited cementum-like matrix adhered to the existing cementum and dentin surfaces, drilled or scraped during surgery (Figure 4).

**Figure 5:**
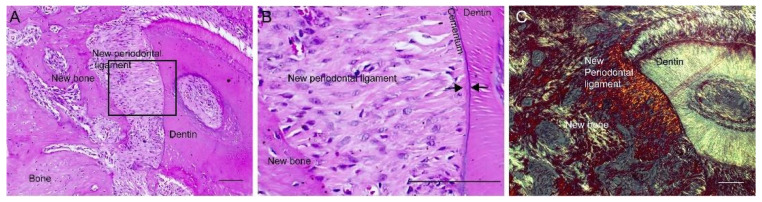
Deposition of cementum-like matrix on dentin and organization of new periodontal ligament on defects without treatment (T0), as thin acellular cementum layer (**A**–**C**). Higher magnification of the indicated area of (**A**), with perpendicularly inserted fiber bundles on the cementum-like matrix (between arrows) (**B**). Periodontal tissue fibers anchored between new bone and cementum-like matrix newly deposited on dentin (**A**–**C**). Polarized PSR imaging of the same specimen (**C**). H&E stain (**A**,**B**); and PSR stain (**C**); scale bars 100 µm.

The newly deposited cementum-like matrix was kept intact without the artifact of tissue separation from histological preparation. The amniotic membrane adjacent to the existing cementum and dentin supported the deposition of the cementum-like matrix, observed as thin acellular layers (Figure 4A–C) or in cellular cementum agglomerates (Figure 4B). The amniotic membrane separated from the existing cementum and dentin, creating a periodontal healing space, supported the regeneration of the periodontal ligament structure (Figure 4C). Periodontal regeneration was observed through collagen fiber bundles that made up the new periodontal ligament inserted perpendicularly into the newly deposited cementum-like tissue (Figure 4C and Figure 5). Periodontal fibers were anchored on both sides on the newly deposited bone and cementum-like matrix (Figure 4C and Figure 5).

Defects without treatment also presented bone healing (T0, Table 1) and periodontal regeneration, with periodontal ligament fibers connected between the newly deposited bone and cementum-like matrix, on the drilled dentin/cementum surface (Figure 5A–C).

## 4. Discussion

Based on the observations of this study, DAM is a potential scaffold in tissue engineering applications as a carrier of cells and ECM. DAM was an appropriate cell carrier, ensuring cell attachment, proliferation, and differentiation during an in vitro period, as expected for grafting. Scaffolds prepared with DAM, ASCs, and ECM were tested in vivo in furcation defects in rats between the roots of the upper first molars compared to control without treatment. The transplantation of DAM, ASCs, and ECM in the defects did not hinder or present adverse effects on the bone healing process, where DAM supported the neovascularization and “conducted” the migration of endogenous osteoblastic cells into the defect. Instead of gradual resorption, DAM was integrated with the deposited tissue concomitantly with bone deposition [32]. Vascularization, cell proliferation, and cell migration from the bone margins of the defect and along the dental root were followed by bone and periodontal regeneration in the narrow space for the new periodontal ligament organization, as described [44].

The decellularization method employed in the preparation of the DAM did not impair ASCs adhesion, proliferation, and differentiation. It preserved the fibrillar membrane structure during the in vitro culture period of one month and as a bone graft for another month. The DAM provided stability to cells, preserving the structural integrity for transplantation afterward with extensive flexibility to be stretched, folded, or rolled. Characteristics that favor manipulation and adaptation are considered advantageous for the application of amniotic membranes in tissue engineering strategies [28,30,36,37,45]. The research on cell delivery strategies is essential because cell transplantation is a potential approach in achieving periodontal tissue regeneration through direct cell participation or paracrine stimulation of the process [5]. Monolayers or cell sheets can be prepared with cells and ECM as alternatives for autologous or allogenic bone grafts, associated or not with the membrane or framework [7,46]. Cell impact was demonstrated in other studies with a periodontal ligament stem cell monolayer transferred onto an amniotic membrane, which presented more bone and periodontal regeneration compared to the membrane without cells [36,37].

The osteoinduction medium promoted ASCs differentiation on DAM with the deposition of the mineralized extracellular matrix on the collagenous fibers of the DAM, creating a transplantable three-dimensional framework with ECM and cells. The deposition of mineralized globular accretions [32] corroborates with the mineralized matrix secreted by differentiating osteoblasts on polystyrene and titanium, explaining the osteoconduction, bone-bonding, and structure of the interfacial matrix [47]. The scaffold material used was crucial for ASCs and ECM retention in the transplantation and oriented bone deposition. As an interfacial matrix between old and new bone, the presence of the reversal line demarcated the pattern of bone deposition in the furcation defect and demonstrated the osteoconduction of the amniotic membrane collagenous matrix. The collagenous niches for osteogenesis were confirmed between the DAM folds and between loose collagenous fibers of the DAM, as observed previously in multilayered DAM [32].

The resulting scaffold associated with the mineralized extracellular matrix by the differentiated ASCs provided osteogenic proteins, such as OPN, as reported by others [48,49]. The pre-osteoinduction of transplanted cells stimulated ECM deposition, cell differentiation [50], and guaranteed the preparation of cell sheets for clinical transplantation [7].

The mineralization of collagen scaffolds stimulated cell proliferation and differentiation in vitro, and bone regeneration in calvarial defects [51], with a higher bone regeneration with cell-seeded mineralized DAM, compared to cell-seeded non-mineralized DAM [49]. The DAM with mineralized ECM did not impair osteoconduction and the presence of the mineralized tissue and bone proteins may even promote osteoinduction [49,52,53], directing endogenous cell differentiation.

Periodontal regeneration was observed in defects treated with DAM, with the cementum-like matrix deposited adjacent to the grafted membrane, bone formed from the DAM, supporting the newly established periodontal ligament and the new periodontal ligament inserted perpendicularly into the cementum-like tissue. It was described that treatments which preserve the natural endogenous periodontal response space presented more regeneration than those with tissue collapse close to the dental roots because of the compression of the treatment or its exposure [3,44]. Bone healing and periodontal regeneration, with cementum deposition and periodontal ligament organization were also observed in the present study in defects without treatment (control). Authors reported the growth of new cementum on and from pre-existing cementum adjacent to the undisturbed periodontal ligament [1,10] in the control group in dogs [54] and a furcation defect in primates implanted with a collagenous matrix [10]. The retention of the blood clot and the granulation tissue responsible for neovascularization inside the self-contained defect anatomy may provide a matrix for the progenitor and stem cell migration from the surrounding periodontal ligament tissue, resulting in periodontal regeneration [3] without any graft treatment in the animal/defect model used in the present study.

Besides the isolation from other faster-repairing tissues, the mechanical stability of early healing tissues and grafts is essential for bone and periodontal regeneration, which is promoted by anchorage-dependent cells [55]. The lack of stabilization of mucogingival flaps and any rupture of the wound closure impairs the establishment of a space for coronal tissue growth and the regeneration of bone and periodontal tissues, encouraging migration and the progressive proliferation of the gingival epithelial tissues and the formation of a long junctional epithelium [44]. The stability of the mucogingival flap of defects was decisive for the results observed with a higher bone healing density in T1 and T2 compared to control (T0) on the most gingival level analyzed (Level A). The grafts with ECM (T3 and T4) did not present a significant difference to T0 on the most gingival level. The possible increase in volume and stiffness of grafted scaffolds with EC due to the presence of calcified globular accretions [32] as a limiting factor to mucogingival flap adaptation to root surface was observed by other authors [56].

This study was unable to demonstrate the ASCs and ECM effect in the class II furcation periodontal defect treatments in rat maxilla with DAM as a bone graft material. It was emphasized that the DAM also, per se, was able to promote osteoconduction and deliver cells based on its collagen structure as scaffolds.

Material and defect shape selection is essential for developing cytotherapeutic approaches in periodontal regeneration [7]. The mucogingival incision healing by the first intention, which must exhibit complete closure, resistance to masticatory forces, and adaptation of the flap [44], is a challenge in the rat periodontal furcation defect model by the presence of the avascular rigid root surface. Discrepancies could be attributed to defects with gingival access without a perfect gingival sealing, affecting graft stability and enabling the apical growth of the gingival epithelial tissue. Further research should be undertaken with another defect model to investigate the effect of cells and the mineralized extracellular matrix on periodontal regeneration. A mandibular fenestration defect in rats is considered a better choice of periodontal model to validate tooth-supporting regenerative therapies [37,57].

Transplanted labeled cells were observed in regenerated periodontal tissues four weeks after transplantation [37]. The detection of transplanted ECM and ASCs was not addressed in this study, but is essential to identify the matrix localization and cell fate to clarify their participation in the healing process.

## 5. Conclusions

The decellularized amniotic membrane with adipose-derived stromal cells or without cells and the mineralized extracellular matrix ensured bone tissue healing. The membrane supported neovascularization and promoted osteoconduction.

## Figures and Tables

**Table 1:** Bone healing percentage (%) of control (T0) and four different graft treatments with DAM, ASCs, and ECM (T1 to T4), on five levels of furcation defects (A–E).

	Treatments					
Level	T0	T1	T2	T3	T4	*p* *
	(*n* = 20)	(*n* = 5)	(*n* = 6)	(*n* = 4)	(*n* = 5)	
A Gingival	19.4 ^a^ ± 17.2	49.1 ^b^ ± 27.4	49.8 ^b^ ± 27.8	27.4 ^a,b^ ± 33.7	24.7 ^a,b^ ± 17.0	0.006
B	29.5 ± 27.6	53.4 ± 24.5	58.0 ± 28.6	39.0 ± 37.3	32.4 ± 14.6	0.088
C	38.2 ± 28.9	67.9 ± 21.5	66.5 ± 27.5	50.3 ± 33.8	32.6 ± 16.5	0.077
D	45.4 ^a^ ± 29.1	77.5 ^a^ ± 15.5	72.0 ^a^ ± 22.8	58.3 ^a,b^ ± 29.4	33.1 ^b^ ± 13.9	0.039
E Apical	52.3 ± 27.7	82.8 ± 11.0	75.8 ± 19.4	69.3 ± 26.8	38.5 ± 19.0	0.06

Data expressed as mean ± sd. *: By Repeated Measures of ANOVA. Data were arcsine square root transformed to improve normality. Means with different letters are significantly different (Student–Newman–Keuls test).

## Data Availability

Not applicable.
